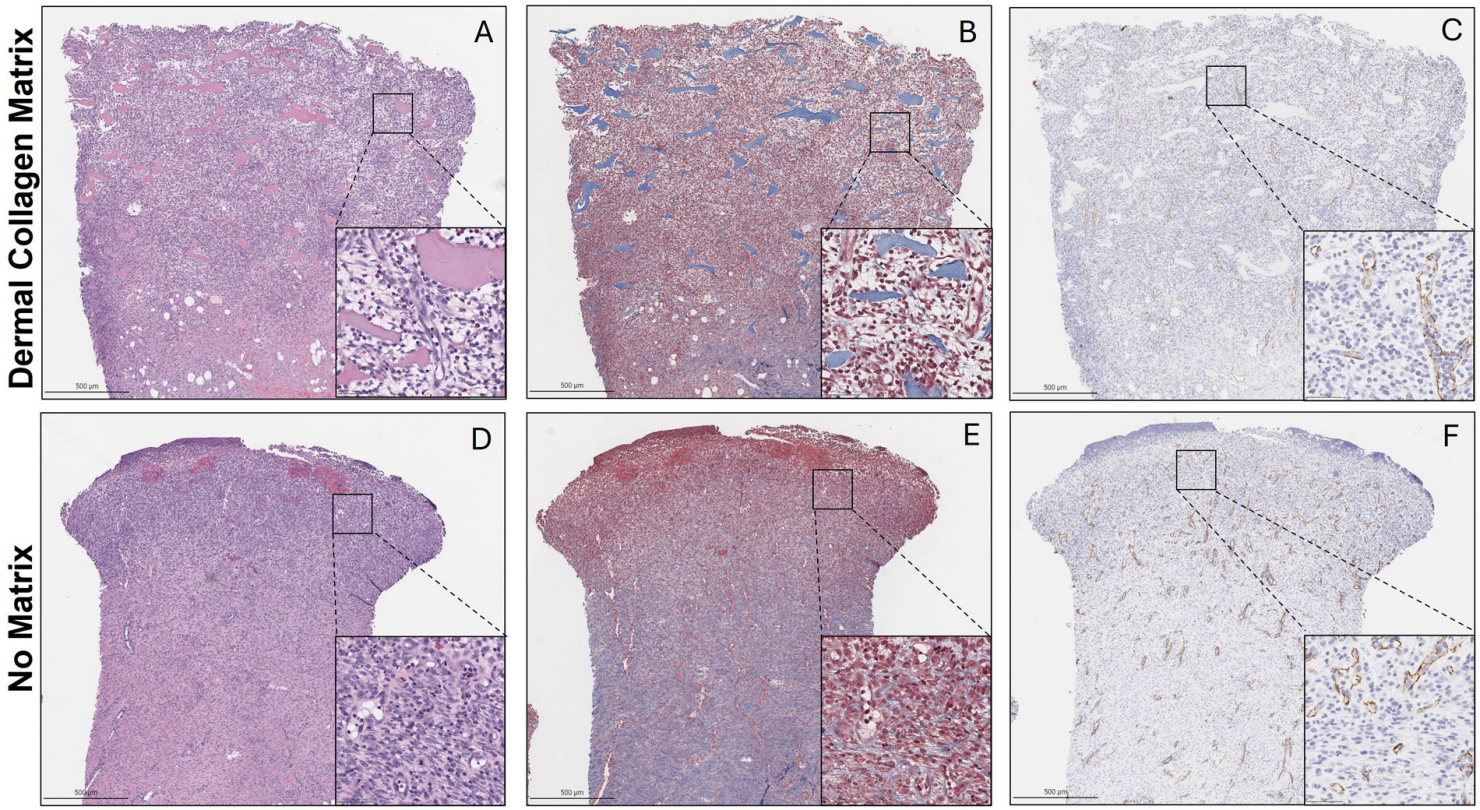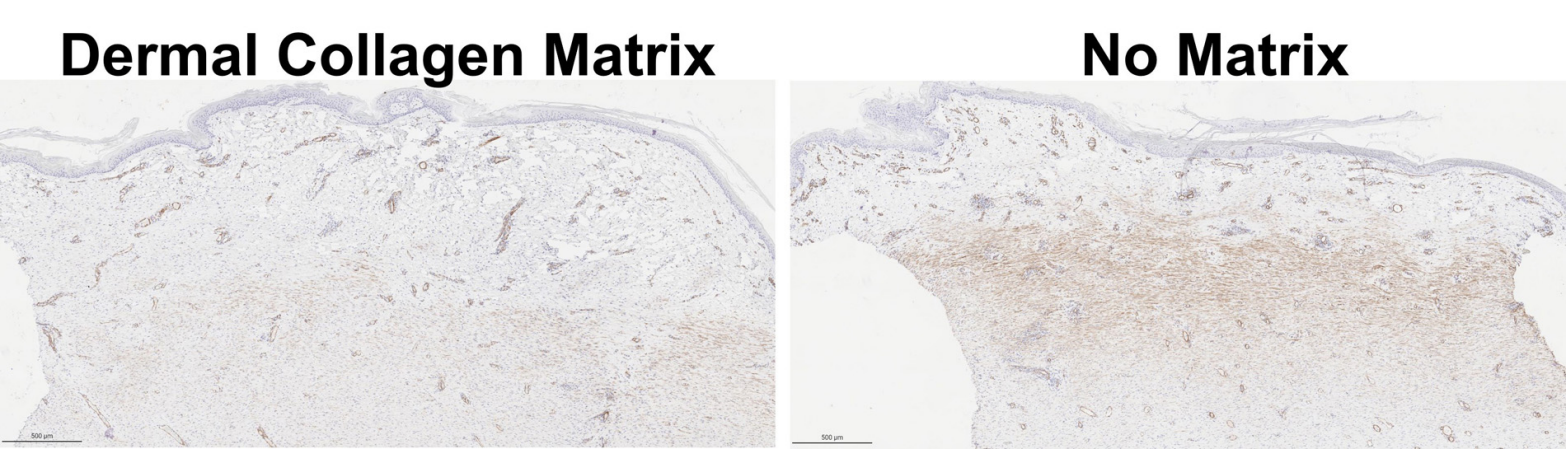# 819 A Bovine Collagen Dermal Matrix Modulates and Improves Dermal Remodeling in Porcine Full-thickness Wounds

**DOI:** 10.1093/jbcr/iraf019.350

**Published:** 2025-04-01

**Authors:** Aleisha Chamberlain, Barbara Nsiah, Jayson Jay, Rachel Penny, Sohail Jahid, Ghaidaa Kashgari, Niraj Doshi, Katie Bush

**Affiliations:** AVITA Medical; AVITA Medical; AVITA Medical; AVITA Medical; AVITA Medical; AVITA Medical; AVITA Medical; AVITA Medical

## Abstract

**Introduction:**

A bovine-derived dermal collagen matrix (DCM) was developed and is currently under review by the FDA for the management of full-thickness cutaneous wounds. The material hydrates quickly and conforms to the wound bed; pores within the 3-dimensional structure facilitate rapid absorption of blood, seeding the matrix with the patient’s own cells and growth factors. This study evaluates tissue formation and remodeling response following delayed autografting in wounds treated with DCM compared to no DCM.

**Methods:**

Full-thickness excisional wounds (16 cm2) were created bilaterally on the dorsum of Yorkshire pigs. Wounds were randomly assigned treatment with DCM or no matrix (n=8 each). On day 7, wounds were biopsied, debrided to obtain punctate bleeding, and grafted with a 3:1 meshed autograft (0.010”-0.012”) and autologous skin cell suspension. Wounds were visually assessed and scored for matrix integration, autograft take, re-epithelialization, inflammation, infection, and contraction. Biopsies were collected on day 7 and day of euthanasia. Histopathological evaluation and analysis were performed to assess inflammation, immune response, blood vessel formation, and alpha smooth muscle actin (αSMA) expression. αSMA-positive cells were quantified using ImageJ (NIH) to determine the percent area expression in the first 2 mm depth of tissue.

**Results:**

The DCM was well-integrated throughout the wound beds with homogenous cell infiltration observed throughout the developed tissue. Histological observation indicated fibrillar-like collagen present, compared to loose collagen deposition indicative of granulation tissue. Blood vessels formed throughout the entire wound bed for both groups (Figure 1). Histopathological scoring of inflammatory cells revealed no significant difference between groups. Similar skin graft take rates were observed with and without the DCM, 96.88 ± 7.04% and 98.13 ± 3.72%, respectively. At day 42, wounds treated with the DCM had significantly reduced αSMA presence (p=0.0035) compared to non-treated wounds (Figure 2). This finding correlated with significantly reduced contraction (p=0.0352), with DCM-treated wounds contracting 7.16 ± 14.08% and non-treated wounds contracting 34.98 ± 8.99% at day 42 (35 days post-autograft).

**Conclusions:**

DCM promotes formation of healthy vascularized tissue which supports excellent autograft take rates at 7 days in a porcine full-thickness wound model. Additionally, the DCM provides an environment which may have the capacity to modulate myofibroblast cell infiltration and impact healing outcomes.

**Applicability of Research to Practice:**

Use of dermal matrices in full-thickness wounds have the potential to modulate cellular integration and remodeling responses, serving as a tool to improve patient outcomes.

**Funding for the Study:**

AVITA Medical